# Social Integration Moderates Longitudinal Links Between Health Constraints and Perceived Stress in Older Adults

**DOI:** 10.1093/geroni/igaf122.2078

**Published:** 2025-12-31

**Authors:** Laura Buchinger, Johanna Drewelies, Valentin Vetter, Ilja Demuth, Denis Gerstorf

**Affiliations:** Humboldt-Universität zu Berlin, Berlin, Berlin, Germany; Max Planck Institute for Human Development, Berlin, Berlin, Germany; Charité - Universitätsmedizin Berlin, Berlin, Berlin, Germany; Charité - Universitätsmedizin Berlin, Berlin, Berlin, Germany; Humboldt University Berlin, Humboldt University Berlin, Berlin, Germany

## Abstract

Stress is a normative experience that likely imposes considerable individual and societal costs due to its strong links with accelerated aging, early retirement, and numerous health conditions. As life expectancy increases, more individuals reach advanced ages that are often characterized by declining physical abilities and social resources. These age-related changes can in turn heighten stress vulnerability, making effective coping more and more difficult. In this preregistered study, we applied longitudinal multilevel and bivariate growth curve models to investigate associations of trajectories of four (performance-based) measures of physical health (morbidity, frailty, grip strength, and the timed-up-and-go (TUG) test) with trajectories of perceived stress. We examined social integration as a potential moderator. Longitudinal data from the Berlin Aging Study II (N = 1,598, age range: 60–94) span 11 years, with perceived stress assessed ten times and physical health assessed three times during this period. Findings indicate statistically significant between-person and within-person associations. For example, steeper increases in frailty and more pronounced declines in the TUG test are each associated with steeper increases in perceived stress. Results also revealed that social integration moderated within-person effects of frailty and TUG performance on perceived stress, thereby alleviating the negative impact of health constraints on stress. Our findings underscore the interplay between physical health and stress in old age, highlight the protective role of social integration, and emphasize the need for interventions that enhance social resources to support well-being in later life.

